# Continuous Ventricular Volumetric Quantification in Patients with Arrhythmias using Real-Time 3D CMR-MOTUS

**Published:** 2026-03-04

**Authors:** Thomas E. Olausson, Maarten L. Terpstra, Rizwan Ahmad, Edwin Versteeg, Casper Beijst, Yuchi Han, Marco Guglielmo, Birgitta K. Velthuis, Cornelis van den Berg, Alessandro Sbrizzi

**Affiliations:** 1 Computational Imaging Group for MRI Therapy & Diagnostics, Center of Image Sciences, University Medical Center Utrecht, Utrecht, the Netherlands; 2 Department of Biomedical Engineering, The Ohio State University, Columbus, United States of America; 3 Department of Radiotherapy, University Medical Center Utrecht, Utrecht, the Netherlands; 4 Division of Cardiovascular Medicine, The Ohio State University, Columbus, Ohio, United States of America; 5 Department of Radiology, University Medical Center Utrecht, Utrecht University, Utrecht, the Netherlands; 6 Department of Cardiology, University Medical Center Utrecht, Utrecht University, Utrecht, the Netherlands

**Keywords:** Free-Running, Real-Time, Motion fields, Arrhythmia, Premature ventricular contraction, Beat-to-beat variability, Volumetric quantification, Ejection fraction

## Abstract

**Background:**

Conventional cardiovascular magnetic resonance (CMR) cine sequences rely on binning reconstructions that average multiple heartbeats, an assumption that breaks down in arrhythmic patients where beat-to-beat variations lead to motion artifacts and loss of clinically relevant functional information. While 2D real-time imaging can capture individual heartbeats, a stack of 2D slices is sub-optimal to map the full complexity of incoherent cardiac dynamics during arrhythmia. We demonstrate the feasibility of 3D real-time motion field reconstruction for continuous beat-to-beat volumetric quantification in patients with premature ventricular contractions (PVC) using a free-running CMR protocol.

**Methods:**

We extended CMR-MOTUS to jointly reconstruct real-time 3D motion fields and a motion-corrected reference image from continuously acquired data without breath-holds or ECG gating. A variable-density Cartesian sampling trajectory (OPRA) was used with a 3D spoiled gradient echo or balanced steady-state free precession sequence. The real-time volumetric beat-to-beat changes were quantified by propagating a single manual segmentation on the reference image, through all time frames using the reconstructed motion fields. The method was validated on a cardiac motion phantom with ground-truth static acquisitions and tested in 4 healthy volunteers and 4 patients with PVC. The ejection fraction (EF) was compared to ground-truth values for the phantom and to standard 2D real-time cine EF measurement techniques for in-vivo subjects.

**Results:**

Reconstructed EF values of the phantom experiment showed good agreement with the ground-truth(EF = 22.1 ± 0.6% versus 21.9%). In healthy volunteers, the mean EF values were close to 2D reference measurements and narrow beat-to-beat EF distributions reflected normal physiological consistency. In PVC patients, the method revealed bimodal EF distributions, with the lower mode corresponding to PVC episodes where individual beats had substantially reduced ejection fractions. Simultaneously acquired ECG signals confirmed the temporal correspondence between volume irregularities and PVC episodes.

**Conclusions:**

3D real-time joint motion field and image reconstruction from a free-running CMR protocol enables continuous beat-to-beat volumetric quantification in arrhythmic patients, revealing functional heterogeneity that conventional single-beat and averaging measurements (binning and gating) obscure. The bimodal EF distributions observed in PVC patients quantify the true hemodynamic impact of arrhythmic episodes and may provide clinically relevant metrics for treatment monitoring and outcome prediction.

## Background

Cardiovascular MRI (CMR) is the gold standard for non-invasive volumetric assessment of cardiac structure and function.[1] Traditional volumetric indicators such as ejection fraction (EF), end-diastolic volume (EDV) and stroke volume (SV) are widely used to quantify cardiovascular diseases.[2] However, these global parameters lack regional information and therefore fail to detect regional abnormalities despite normal EF, EDV and SV values.[2], [3], [4] A study has demonstrated the added clinical value in quantifying subtle regional motion variations using deformable image registration (DIR) to monitor progression and responses to treatment of cardiovascular diseases.[2] Other studies show that DIR can provide strain, strain rate, and velocity measurements while enabling automated computation of the standard volumetric quantities (i.e., EF, EDV, SV) through a one-stop CMR post-analysis workflow.[5]

In order to accurately describe regional function, CMR cine images need to be of high quality with respect to spatiotemporal resolution and SNR. However, due to the inherently low sampling rate of MRI, standard CMR cine sequences rely on binning reconstructions that average multiple heartbeats during the acquisition to achieve acceptable image quality.[6] While various motion surrogate signals and regularization strategies have been developed to improve binning methods, they fundamentally assume near identical cardiac cycles throughout the acquisition.[7], [8], [9] This assumption breaks down for arrhythmic patients, where beat-to-beat variations in morphology and function lead to motion artifacts and degrading image quality.[6], [10] Some approaches discard data acquired during arrhythmic episodes (arrhythmia rejection), but this reduces the acquisition efficiency when arrhythmia is frequent and loses valuable functional information during these arrhythmic events.[11] Additionally, respiratory motion can influence the hemodynamics and morphology of the heart.[12] Currently the clinical CMR methods for arrhythmia rely on 2D real-time MRI to capture these changes during breathing.[13] These methods ensure volumetric coverage by acquiring a stack of 2D images in rapid succession. However, using a stack of 2D imaging is sub-optimal to map the complexity and incoherence of heart dynamics, as the arrhythmic beats are captured on some slices but not others. Hence, 3D real-time imaging may enable the accurate measurement of cardiac hemodynamics and morphology, including the effects of arrhythmias.

Real-time MRI offers an alternative to binning methods by continuously acquiring data and reconstructing each heartbeat separately, visualizing the true physiological cardiac morphology in real time .[10] However, the high spatiotemporal requirements to measure real-time cardiac dynamics forces high undersampling of the MRI acquisition (R>10). Several methods have proposed to address the extreme undersampling, using innovative acquisition and reconstruction techniques. However, many rely on challenging non-Cartesian k-space sampling trajectories that can produce eddy current artifacts and require lengthy reconstruction times due to repeated gridding operations.[14], [15]

Recently, the ML-DIP framework successfully used a temporally incoherent variable density Cartesian sampling for 3D real-time reconstruction at a high temporal resolution, capturing beat-to-beat variability in arrhythmic patients. However, the reconstructed images components in ML-DIP are not guaranteed to be motion-static, meaning the motion fields may not fully capture the motion dynamics, limiting their clinical interpretation[16]. Separately, CMR-MOTUS demonstrated a framework for jointly reconstructing real-time cardiorespiratory motion fields and contrast-varying motion-static images using low-rank assumptions, achieving promising 2D results with both Cartesian and non-Cartesian sampling.[17] This disentanglement of physiological motion in CMR-MOTUS with motion fields could enable a comprehensive cardiac functional assessment and the continuous beat-to-beat volumetric analysis workflow in arrhythmic patients.

In this study, we aim to achieve real-time 3D imaging and motion field characterization. To achieve this, we extend CMR-MOTUS to 3D using an efficient Cartesian sampling pattern to reconstruct real-time cardiovascular motion fields at 20 Hz. Our approach leverages explicit, interpretable regularization strategies rather than an implicit deep learning regularization, employing a low-rank motion field model without convolutional neural networks. This reduces the number of optimizable parameters which leads to faster reconstructions. The co-reconstruction of 3D+t motion fields inherent to CMR-MOTUS simplifies downstream tasks such as real-time segmentation propagation for volumetric quantification in arrhythmic patients who would otherwise be ineligible for standard analysis. Due to the absence of in-vivo 3D+t ground truth (GT) data, we validate the reconstructed motion fields and volume quantification using a cardiac insert in a motion phantom[18]. In addition, we analyse data from 4 healthy volunteers and four arrhythmic patients and compare our extracted volumetric quantification parameters to the values derived from 2D imaging using clinically accepted software.

## Methods

### Framework

CMR-MOTUS jointly solves for a motion-corrected reference image and real-time deformation vector fields by solving the following minimization problems

(1) $$\underset{q}{min}\sum_{t}^{M}{\left\|F\left(q|D_{t}\right)-s_{t}\right\|}_{2}^{2}$$

(2) $$\underset{\left[D_{1},\ldots ,D_{M}\right]=\Phi \Psi^{T}}{min}\sum_{t}^{M}{\left\|F\left(D_{t}|q\right)-s_{t}\right\|}_{\ell }+TV(D)$$

where q represents the motion-corrected reference image, $D_{t}$ are the real-time motion fields at each of M time points, $s_{t}$ is the acquired k-space data at time t, and F denotes the forward encoding operator that includes coil sensitivity multiplication, Fourier transform, k-space sampling mask, and the backwards warping operation.

The motion fields are parameterized as dense displacement vectors, where each voxel is assigned a 3D displacement vector. To reduce memory requirements as in Low-Rank MR-MOTUS, we use an explicit low-rank factorization $D=\Phi \Psi^{T}$, where $\Phi \in R^{Nd\times R}$ contains R spatial basis components for N voxels across d=3 spatial dimensions, and $\Psi \in R^{M\times R}$ contains the corresponding temporal basis components. This explicit factorization avoids computing singular value decompositions (SVD) during optimization in the implicit factorization case, which would be computationally expensive for 3D data at 20 Hz temporal resolution. The full motion field $D\in R^{Nd\times M}$ is recovered through matrix multiplication $D=\Phi \Psi^{T}$, substantially reducing the number of parameters compared to directly optimizing all displacement vectors across time.

The data consistency term uses different norms depending on the dataset characteristics. For phantom data, an $\ell_{2}$ norm is used as these acquisitions contain minimal outliers. For in vivo data, an $\ell_{1}$ norm is used to provide robustness against localized inflow effects caused by blood entering the imaging volume, which should not affect the motion fields.[19] The regularization term TV(D) applies isotropic total variation (TV) in the spatial domain to promote spatially smooth motion fields while preserving sliding motion.[20] The regularization weight λ is scaled relative to the data consistency, enabling comparable weight selection across different datasets. The specific value of λ was selected empirically through a parameter sweep on phantom data as described in the data analysis section and kept the same values for the in-vivo data.

We adjusted the CMR-MOTUS image reconstruction step to only reconstruct a time-independent reference image, instead of a time-dependent reference image. This modification distinguishes our 3D implementation from the original 2D CMR-MOTUS framework[17], which used a low-rank plus sparse decomposition to model temporal contrast variations. While that decomposition effectively captured inflow effects in 2D, extending it to 3D at 20 Hz would require computing singular value decompositions on matrices with thousands of frames at each iteration, making the approach computationally expensive. By reconstructing a single motion-corrected reference image, in combination using the $\ell_{1}$ norm for in vivo data and isotropic TV regularization in motion fields, we achieve similar robustness to inflow effects with substantially reduced computational cost. Additionally, all data used in this study have a time stable contrast as there are no contrast agent inflow effects, and the use of steady-state acquisitions.

### Data Acquisition

#### K-Space Sampling Trajectory

All following experiments used the Ordered Pseudo RAdial (OPRA) k-space sampling trajectory[21]. For this Cartesian sampling trajectory, we acquired readout lines along the FH direction. In the axial plane, readouts are sequentially acquired along an “L”-shaped leaflet which rotates in the plane using the golden angle. At lower spatial frequencies, spacing between samples is reduced compared to higher spatial frequencies resulting in a variable density in the axial plane. We used the default settings of this sampling trajectory as described in[21], with an exception to the k-space matrix size.

#### Phantom Study

**Figure 2:**
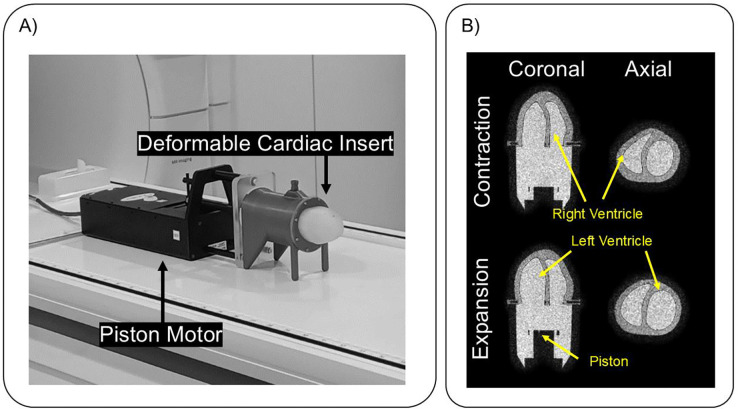
Illustration of phantom anatomy. (A) shows the experimental set up of the cardiac phantom on the MRI table. The phantom is a silicone based deformable insert with two compartments to mimic the right and left ventricle of a human heart. Liquid inside the compartment is pushed by a piston which makes the phantom expand and contract. Due to the design of the phantom, the right ventricle has a 10% higher ejection fraction than the left ventricle. Hence for our experiment we used right ventricle for analysis. (B) shows the 3D reference images acquired when the piston is static at largest displacement (expansion) and lowest displacement (contraction). These images were later used to segment the right ventricle and determine the ground truth ejection fraction. The yellow arrows label the anatomy of the phantom.

We validated the proposed approach using a cardiac insert[18] for the Modus QA motion phantom (IBA Dosimetry GmbH, Schwarzenbruck, Germany). In short, the deformable phantom is driven by a piston which compresses the liquid into two expandable silicon containers, which are shaped to mimic the left and right ventricles of a human heart. Figure 2A shows the experimental setup on the MRI table with the phantom. The silicon material and liquid have similar relaxation properties to myocardium and blood respectively (Left ventricle: T1 = 763 ± 76 ms, T2 = 49 ± 12 ms. Right ventricle: T1 = 775 ± 69 ms, T2 = 185 ± 35 ms. Blood: T1 = 1130 ± 44 ms, T2 = 155 ± 16 ms.) For the maximum piston motion amplitude achievable (and EF) with this phantom design, we operated the motion phantom at 60 oscillations per minute using a simple sinusoidal function.

All phantom acquisitions were performed on a 1.5T MRI scanner (Philips Ingenia, Philips Healthcare, Best, The Netherlands). We used a 3D T1-weighted spoiled gradient echo sequence with the following parameters: TR/TE = 2.4/1.07 ms, flip angle = 6°, field of view = 117 × 173 × 120 mm^3^, spatial resolution = 2.00 × 2.00 × 2.00 mm^3^. Total scan duration was approximately 96 seconds. The phantom piston operated continuously at 60 oscillations per minute throughout each acquisition.[22] For ground-truth validation, we acquired two static 3D images with the motionless piston at its peak positions (maximum and minimum displacement, respectively). These reference acquisitions used the same sequence parameters as the dynamic acquisitions but employed fully sampled linear Cartesian k-space sampling. Figure 2B shows images from these reference acquisitions with the inner anatomy of the phantom labelled.

#### In Vivo Study

All in vivo studies were approved by the institutional review board, and written informed consent was obtained from all participants. We tested the method in vivo with two subject groups: healthy volunteers and patients with premature ventricular contraction (PVC). The PVC group consisted of 3 females and 1 male (age 37 ± 17 years, BMI 22.5 ± 3.2 kg/m^2^), while the control group consisted of 2 females and 2 males (age 36 ± 8 years, BMI 25.3 ± 4.0 kg/m^2^). Imaging was performed on two 1.5 and 3 Tesla MRI systems (MAGNETOM Sola and MAGNETOM Vida, Siemens Healthineers, Erlangen, Germany). PVC patients were scanned on the Sola system using a 3D balanced steady-state free precession (bSSFP) sequence approximately 20 minutes after the administration of a gadolinium-based contrast agent. Healthy volunteers were scanned on the Vida system using a 3D gradient echo sequence post ferumoxytol contrast enhancement. Imaging parameters were: TR/TE = 2.89–3.17/1.22–1.34 ms, flip angle = 14–40°, spatial resolution = [1.4–2.2] × [1.56–1.88] × [1.54–1.64] mm^3^. All acquisitions were performed using a free-running protocol, with free-breathing and no ECG gating. The total scan time for each acquisition was approximately 2 minutes.

End-diastolic volume, end-systolic volume, and EF values were obtained from standard 2D real-time cine short-axis stacks analysed using dedicated cardiac imaging software (SuiteHEART, NeoSoft, Pewaukee, Wisconsin) according to SCMR post-processing guideline [23]. For PVC patients, the arrhythmic beats were not captured consistently in every slice; therefore, the analysis was limited to dominant sinus contractions.

#### Implementation

The following implementation details were used in all experiments in this study: Algorithmic settings were empirically optimized based on an additional volunteer’s acquisition used for protocol development only. The reconstruction framework was implemented in Python using PyTorch for GPU-accelerated optimization and automatic differentiation. Coil sensitivity maps were estimated from the time-averaged k-space data using the ESPiRIT[24] algorithm. The motion-corrected reference image q was initialized with a time-averaged reconstruction of the acquired k-space data. Motion fields were initialized with spatial low-rank components set to zero and temporal components initialized as smooth oscillating functions with random frequencies between 0.5–1 Hz.

The alternating minimization was performed for a maximum of 120 iterations, which was observed to be typically required to reach a plateau in the cost function. Motion field estimation proceeded using a coarse-to-fine pyramid strategy: the first 30 iterations reconstructed motion fields on progressively finer spatial grids, with the remaining 90 iterations operating on the full resolution grid. During optimization of the motion fields (equation 2), the temporal components Ψ were passed through a Butterworth lowpass filter with a smooth cutoff at 4 Hz to enforce temporal smoothness and remove high-frequency noise. Optimization was performed using stochastic gradient descent (SGD) as implemented in PyTorch. For each backpropagation step, 40 temporal frames were randomly sampled to compute the gradient. This batch size was selected empirically to minimize noise in the reconstructed motion fields while maintaining reasonable convergence speed. For all experiments, hyperparameters were empirically tuned for best visual results in the motion warped images.

A total of 40,000 k-space readouts were acquired and reconstructed into real-time frames by grouping 20 consecutive readouts per frame, resulting in 2,000 frames spanning the entire acquisition. For the phantom acquisitions with TR = 2.4 ms, this corresponded to a total temporal span of 96 seconds and an effective frame rate of 48 ms per frame at ~21 Hz. For in vivo acquisitions with TR = [2.89–3.17] ms, the temporal span was approximately [116–127] seconds with frame rates of [58–64] ms per frame [16–17] Hz. For all datasets, a Rank-16 motion model was used.

Reconstructions were performed on a server equipped with an NVIDIA L40S GPU, with typical reconstruction time of approximately 2 hours per dataset.

### Data Analysis

To establish GT phantom data, manual segmentations of the phantom’s right ventricle were performed in each of the two motion-static 3D images acquired at the piston’s peak displacement positions using *3D Slicer*. We selected the right ventricle in the phantom data because it exhibits a factor 2 larger EF compared to the left ventricle (21.9% versus ~10%). The maximum volume segmentation defined EDV and the minimum volume defined end-systolic volume (ESV), from which GT EF was calculated as

$$EF=\frac{\left(EDV\;-\;ESV\right)}{EDV}\times 100\%$$

For both phantom and in vivo dynamic CMR-MOTUS datasets, a single manual segmentation was performed on the reconstructed motion-corrected reference image q using the same method. As with the GT phantom data, the right ventricle was segmented using the 3D motion-corrected image q. For in vivo data, the left ventricle blood pool was segmented in the 3D motion-corrected image q using *3D slicer*. The segmentation mask was propagated through all reconstructed time frames using the real-time motion fields D, enabling beat-to-beat volumetric quantification throughout the acquisition.

Volume curves were extracted from the real-time propagated segmentations, and peaks (EDV) and troughs (ESV) were automatically detected using the *find_peaks* function from the *SciPy* library in Python. For each detected peak, the algorithm identified the subsequent trough to calculate EF for that specific cardiac contraction.

Mean EF and its standard deviation across all detected cycles within each dataset were computed. The primary validation of motion field accuracy was performed by comparing these real-time EF estimates to the GT measurements from the motion-static acquisitions of the same phantom.

For in vivo datasets, the calculated mean EF was compared to clinical standard EF values obtained from conventional 2D real-time cine acquisitions.

## Results

### Phantom Study

**Figure 3:**
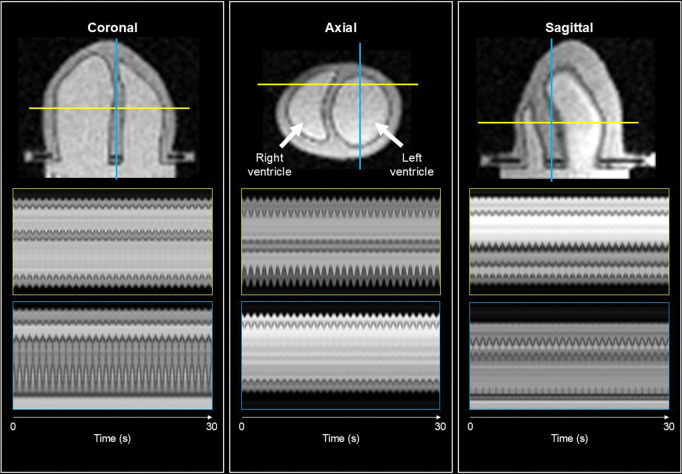
Data shown in the figure is from the real-time phantom acquisition operating at 60 beats per minute. The figure shows the spatiotemporal plots of the real-time cines generated by warping the motion-corrected 3D reference image using the reconstructed motion fields from CMR-MOTUS at ~20 Hz temporal resolution. For each orthogonal view, a red and green line indicates which 1D profiles in the spatiotemporal plots are defined. Below each view are the two spatiotemporal plots corresponding to coloured lines. Across all plots, modulations can be seen for each individual contraction. Note that a 30 second interval is presented here, whilst the entire reconstructed data is roughly 90 seconds long.

Real-time cine images were generated by warping the motion-corrected reference image to each time point using the motion fields reconstructed by CMR-MOTUS. The resulting cine sequence is shown in Figure 3. Using the motion-corrected reference image q, we manually segmented the phantom’s right ventricle. This segmentation was then propagated across all time frames using the reconstructed real-time motion fields to obtain continuous, beat-to-beat volumetric quantification in the right ventricle throughout the acquisition as seen in Figure 6.

The spatiotemporal plots in Figure 3 illustrate the consistency of the motion patterns across multiple cardiac cycles. The modulations visible in each orthogonal view correspond to the periodic expansion and contraction of the phantom. These patterns remained stable across the entire acquisition, suggesting that the reconstructed motion fields accurately capture the phantom’s mechanical behaviour.

The obtained phantom’s ejection fraction was in good agreement with the GT EF determined from the motion-static acquisitions, with the real-time reconstruction obtaining a mean and standard deviation 22.1 ± 0.6% compared to the GT value of 21.9%.

### In Vivo Study

**Figure 4:**
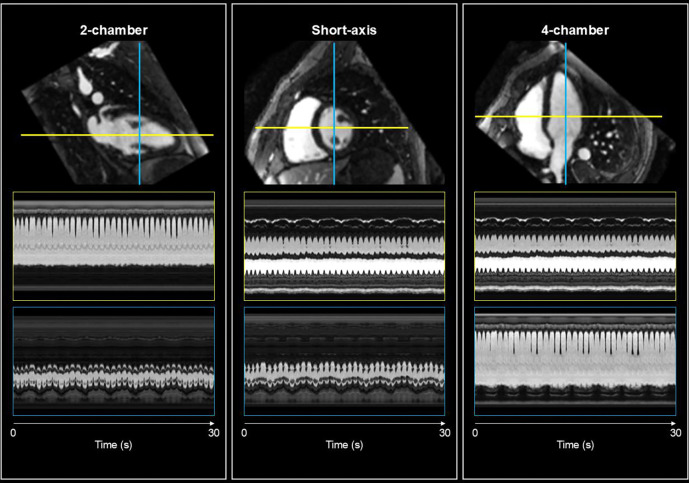
Data shown in the figure is from a single healthy volunteer. The figure shows the spatiotemporal plots of the real-time cines generated by warping the motion-corrected 3D reference image using the reconstructed motion fields from CMR-MOTUS at ~16 Hz temporal resolution. Since the data is isotropic 3D, we reformatted the cine to the standard CMR views (2-chamber, short-axis and 4-chamber). For each view, a red and green line indicates which locations the spatiotemporal plots are defined. Below each view are the two spatiotemporal plots corresponding to coloured lines. Across all plots, modulations can be seen for each individual heartbeat and the breathing cycles. Note that a 30 second interval is presented here, whilst the entire reconstructed data is roughly 2 minutes long.

For all subjects, real-time motion fields and motion-corrected reference images were reconstructed. Figure 4 presents a representative example from a healthy volunteer, illustrating the quality of the reconstructed cine images generated by warping the motion-corrected reference image with the reconstructed motion fields. Since the acquired data is isotropic 3D, we reformatted the reconstructed cine sequences to standard CMR views including the 2-chamber, short-axis, and 4-chamber orientations. This flexibility demonstrates a key advantage of the 3D acquisition approach. The reference image shows minimal motion blurring, and the spatiotemporal plots shows expected cardiorespiratory modulations which both indicate that the reconstructed motion fields are physiologically plausible.

**Figure 5:**
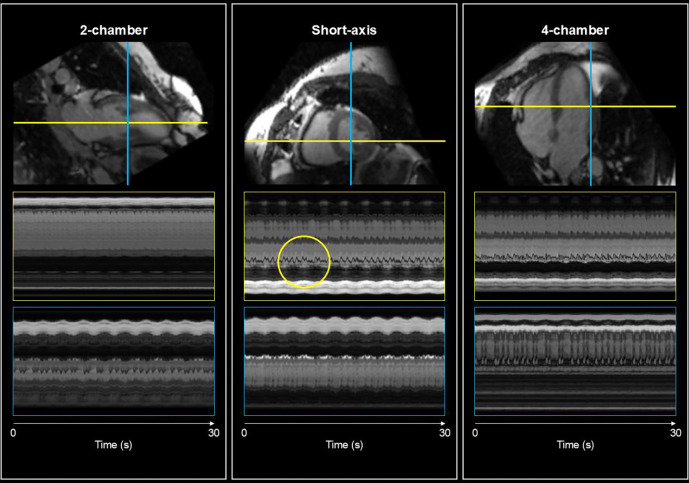
Data shown in the figure is from a single subject with premature ventricular contraction (PVC). The figure shows the spatiotemporal plots of the real-time cines generated by warping the motion-corrected 3D reference image using the reconstructed motion fields from CMR-MOTUS at ~17 Hz temporal resolution. Since the data is isotropic 3D, we reformatted the cine to the standard CMR views (2-chamber, short-axis and 4-chamber). For each view, a red and green line indicates which locations the spatiotemporal plots are defined. Below each view are the two spatiotemporal plots corresponding to coloured lines. Across all plots, modulations can be seen for each individual heartbeat and the breathing cycles. There is irregularity in the modulations due to several PVC episodes highlighting the significant beat-to-beat variability for these patient types. An example of these episodes is circled in yellow in a short-axis spatiotemporal plot. Note that a 30 second interval is presented here, whilst the entire reconstructed data is roughly 2 minutes long.

Figure 5 shows a similar illustration, this time for a patient with PVC. The reference image again shows minimal motion blurring, but now the spatiotemporal plots show highly irregular modulations in the cardiac frequency. These irregularities correspond to the PVC episodes. The varying intervals between consecutive contractions and the differences in contraction patterns are visible in the spatiotemporal plots, highlighting the beat-to-beat variability. This variability would be averaged out in conventional binning-based reconstruction approaches, but the proposed real-time method preserves these clinically relevant dynamics.

**Figure 6:**
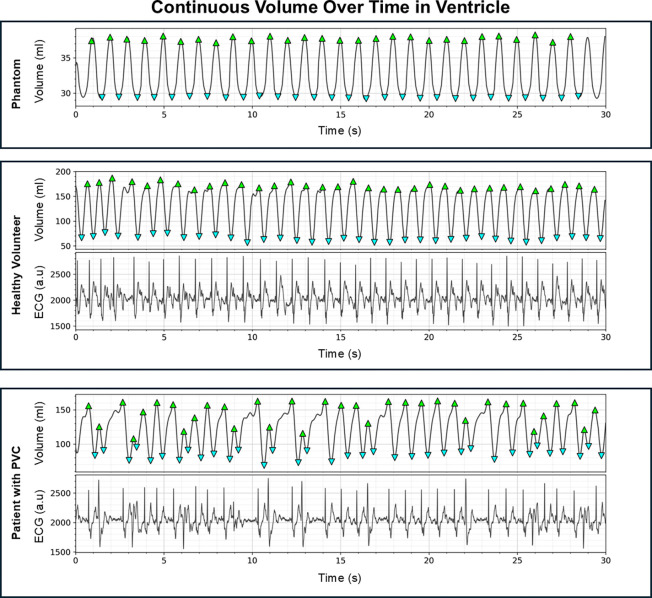
Continuous volume in the ventricle over time plots for the phantom experiment, and a representative subject from the healthy volunteers and patients with premature ventricular contraction (PVC) groups. The temporal resolution for the phantom, healthy volunteer and patient with PVC datasets at around 20 Hz, 16, Hz and 17 Hz respectively. Green and blue triangles represent the peak-trough pairs detected by the find_peaks algorithm in Scipy. Note the beat-to-beat variability in the in vivo cases; most severe in the patient case where there are PVC episodes present. For each peak-trough pair the ejection can be determined giving more understanding of how the ejection fraction varies time, but also how it varies during PVC episodes in the patient case. The plots showcase a 30 second interval of the entire acquisition which is roughly 2 minutes long. For the in vivo cases, the ECG signal that is acquired simultaneously with the acquisition is displayed under the volume plots. This shows that the irregular peak-trough pairs are linked to an irregular signal in the ECG suggesting that this is a PVC event.

By propagating the manually segmented left ventricle through all time frames using the reconstructed motion fields, we obtained continuous volume curves throughout the entire acquisition for all subjects. The propagation process can be seen in Supplementary Figure 1–8for each subject over a short time window. Figure 6 displays a representative volume curve for a healthy volunteer and a patient with PVC. The healthy volunteer shows regular periodic oscillations with consistent peaks and troughs, reflecting stable cardiac function with minimal beat-to-beat variability. In contrast, the patient with PVCs exhibits significant irregularities in the volume curve, with substantial variations in timing of individual cardiac cycles. Some contractions show reduced stroke volumes, corresponding to PVC episodes. Note that the simultaneously acquired ECG signal displayed below the volume curves confirms the correspondence between irregular volume patterns and PVC episodes in the electrical activity. The temporal alignment between irregular ECG and ventricular volume validates that the reconstructed motion fields are capturing true physiological events rather than reconstruction artifacts.

**Figure 7:**
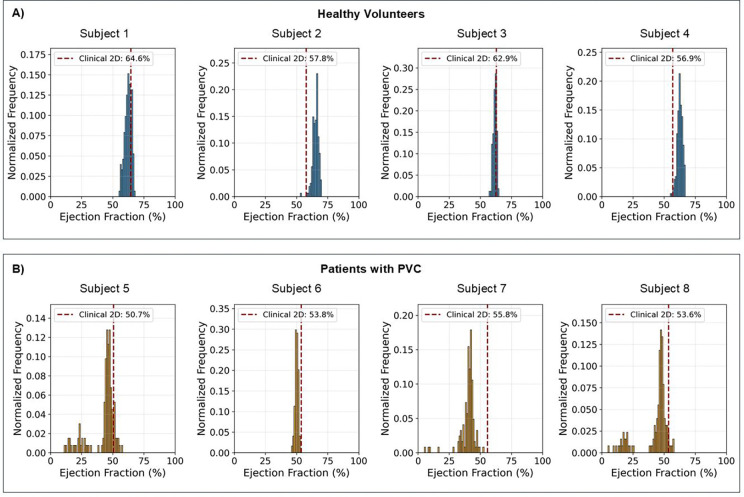
Histogram of the frequency of ejection fractions (EF) determined in the entire ~2-minute in vivo acquisitions with healthy volunteers in A and patients with premature ventricular contraction (PVC) in B. The histogram bin width was set to 1%. A vertical dashed red line indicates the EF determined using the 2D acquisition. There is mixed agreement between the 2D and real-time 3D approaches. What is notable is that the real-time 3D EF offers statistics from the beat-to-beat variability. For subject 6 there were no PVC episodes reconstructed which explains the similar distribution spread as observed in the healthy volunteer cases. For the other patients with PVC, the PVC episodes contribute the to secondary distribution of lower EF as seen particularly in subjects 5 and 8.

Figure 7 presents histograms of beat-to-beat EF distributions across the in vivo acquisitions. For healthy volunteers, the EF distributions are relatively narrow. The slight spread reflects the physiological beat-to-beat variability present even in healthy subjects. This temporal information, absent in conventional averaged acquisitions, provides insight into cardiac function.

For patients with PVCs, the EF distributions show different characteristics. Subjects 5, 7, and 8 show bimodal distributed EF values, with the lower mode corresponding to EF during PVC episodes. These distributions reveal that individual PVC beats can have substantially lower ejection fractions than the patient’s baseline cardiac function. Subject 6, however, displays a narrow distribution similar to healthy volunteers, which is consistent with the absence of PVC episodes during this acquisition. This variability in PVC burden across different acquisitions highlights the clinical value of continuous monitoring throughout the scan.

**Figure 8:**
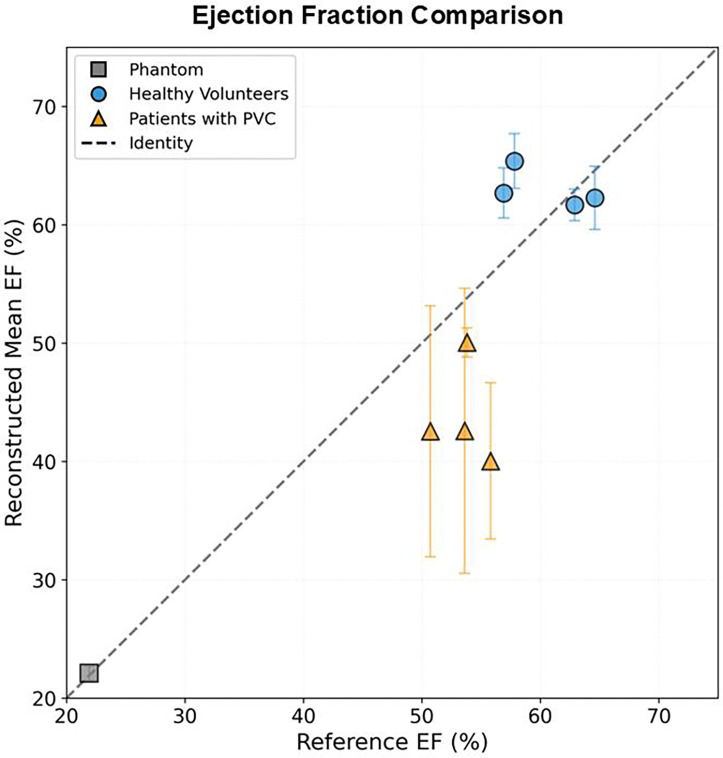
Comparison of the ejection fractions (EF) determined by the proposed continuous 3D volume curve and the standard 2D clinical method in the human subjects. For the phantom study, the reference EF was determined by the motion static acquisitions at different piston amplitudes. Error bars indicate the standard deviation of each measurement. There is good agreement in the phantom study and healthy volunteer studies. However, this is different for the patients with premature ventricular contraction cases. This is attributed to the high beat-to-beat variability that the proposed method captures, as the PVC episodes contribute to an overall lower mean. In one patient (subject 6) without any PVC episodes during reconstruction of the proposed method, the measurements show good agreement.

Figure 8 directly compares the mean EF from the proposed real-time 3D method against the GT and clinical reference values from standard 2D acquisitions. There is good agreement in the phantom and healthy volunteer cases. However, there is poor agreement in the patients with PVC except for subject 6. Notably there were no detected PVC episodes during the acquisition for subject 6, in comparison to the other subjects (5,7,8) who had several PVC episodes.

## Discussion

This study demonstrates the feasibility of a 3D real-time motion field reconstruction in PVC patients using a free-running CMR protocol, enabling automated beat-to-beat volumetric quantification through segmentation propagation. In contrast to other real-time 3D MRI methods, our proposed approach explicitly separates physiological motion into interpretable motion fields and a single motion-corrected reference image, fundamentally enabling a clinical workflow using these motion fields for downstream analysis. We validated this approach using a cardiac phantom and demonstrated its clinical utility by quantifying beat-to-beat functional variability in these patients with PVCs, revealing a bimodal EF distribution (Figure 7) that reflect the true hemodynamic change of PVC episodes.

The beat-to-beat volumetric assessment has potentially important clinical application. First, patients with PVCs may have preserved EF on standard gated CMR but reduced mean function when all beats are considered. Second, therapeutic monitoring after treatment procedures could assess treatment’s effectiveness not only in terms of PVC frequency reduction but also by improvement in EF distribution characteristics and reduction in beat-to-beat variability. Third, the statistical distribution of EF across cycles provides insight into cardiac functional compensatory capacity that one-beat measurements cannot capture. These metrics may prove valuable for guiding treatment decisions and predicting clinical outcomes, though prospective clinical validation studies are needed. On the other hand, the motion fields provide a foundation for comprehensive deformation analysis. Future work could derive desynchrony, strain, strain rate, and regional wall motion metrics from the motion fields to provide complete myocardial functional characterization.

In our phantom validation experiment, the small standard deviation across detected cycles demonstrates the consistency of the reconstruction method. We observed a slight beat-to-beat variation in the reconstructed EF of the phantom, which can be attributed to the different mechanical conditions between the two measurement approaches. In the real-time case, the piston operated continuously, potentially introducing dynamic elastic responses in the silicone material during the expansion and contraction phases. In contrast, the static reference acquisitions were performed at static displacement positions where such mechanical effects were absent. This may contribute to subtle variations in the observed volumes, though the close agreement validates the accuracy of the motion field reconstruction.

There is good agreement between the proposed mean EF and the reference EF (Figure 8in healthy volunteers and the phantom experiments. However, for PVC patients, a systematic underestimation is observed in reconstructed mean EF compared to the 2D reference. This apparent disagreement reflects a fundamental difference in what each method measures. The standard 2D acquisitions (reference EF) uses real-time 2D slice to capture a single heartbeat and then add all the slices together to form one beat. In contrast, the proposed method captures all cardiac events including PVC episodes, resulting in a mean EF that accounts for the reduced function during irregular beats. Subject 6, who showed no reconstructed PVC episodes, demonstrates close agreement between methods, supporting this interpretation.

The underlying framework is not inherently limited to cardiac applications. The same free-running acquisition and CMR-MOTUS reconstruction could in principle be extended to other organs and body regions where physiological motion presents challenges, such as abdominal or head, where speech, respiratory and cardiac motion similarly complicate conventional acquisitions.

### Limitations

This study has limitations. First, the reconstruction time of approximately 2 hours per dataset limits clinical workflow integration. This is comparable to other CMR analyses such as 4D flow, where offline processing is common, though further algorithmic optimization will be needed for routine clinical adoption. The authors have identified several computational solutions which could substantially reduce CMR-MOTUS computation time. This will be left for future studies.

Second, this feasibility study included only 4 healthy volunteers and 4 PVC patients. While this cohort was sufficient to demonstrate technical feasibility of the method and to reveal clinically relevant beat-to-beat variability, it limits the generalizability of the findings. Larger cohort studies are needed to validate reproducibility, and assess the prognostic value of beat-to-beat functional heterogeneity.

Finally, the phantom, healthy volunteers and PVC patients were scanned on different MRI systems with different field strengths, sequences, and contrast agents, which may introduce confounding factors when comparing results between groups. This heterogeneity reflects clinical reality where scan protocols are tailored to the clinical question and available hardware, but future controlled studies should aim for more homogeneous acquisition protocols to isolate the effect of the reconstruction method from acquisition-related variability.

## Conclusion

This study establishes real-time 3D motion field reconstruction as a potentially clinically enabling approach for comprehensive cardiac functional assessment in arrhythmic patients. By explicitly extracting physiological motion into interpretable motion fields, we enable single-segmentation propagation across all cardiac phases, making beat-to-beat volumetric quantification practical and revealing functional heterogeneity that conventional measurements (binning or gating) obscure. In PVC patients, this method quantifies the true hemodynamic changes of arrhythmias. With accurate motion fields validated through phantom experiments and ECG correlation in vivo, this approach provides a foundation for future derivation of desynchrony, strain, strain rate, and regional deformation metrics from a single free-running acquisition.

## Supplementary Material

Supplement 1

## Figures and Tables

**Figure 1:**
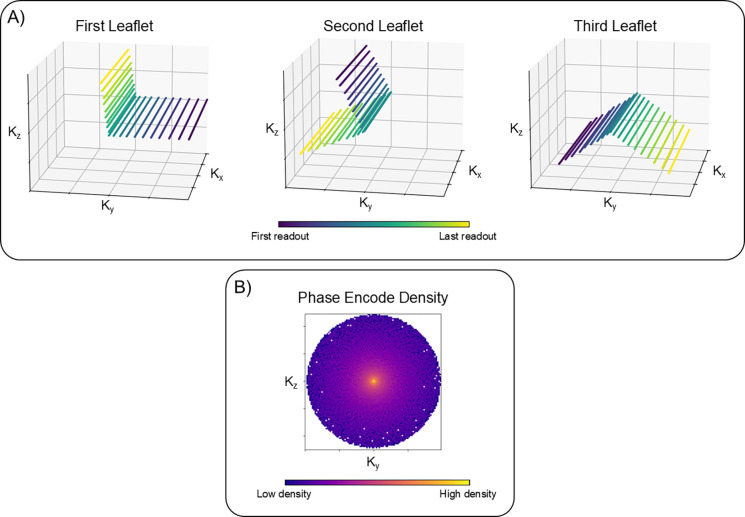
Illustration of the Ordered Pseudo RAdial (OPRA) k-space sampling trajectory using default settings as described in [21]. Readouts are acquired along the kx axis. (A) Shows the k-space samples of the first three frames individually in each subplot. Each subplot contains one of the leaflets at separate golden angle increments. Note the small consecutive jumps between each readout which should minimize the effects of eddy currents. (B) Shows the cumulative sum of total number of readouts in the entire acquisition. Note the variable density in the ky-kz plane where lower spatial frequencies are sampled more often than higher spatial frequencies.

**Table 1:** Summary of MRI acquisition parameters for phantom validation and in vivo studies.

Imaging Parameters	Phantom	Healthy Volunteers	Patients
Scanner	Philips Ingenia, 1.5 T	Siemens MAGNETOM Vida, 3 T	Siemens MAGNETOM Sola, 1.5 T
Sequence	3D T1w spoiled GRE	3D GRE with ferumoxytol	3D bSSFP
Flip angle (°)	10	[14–18]	[32–40]
TR/TE (ms)	2.4/1.07	[3.10–3.17]/[1.22–1.24]	[2.89–3.03]/[1.28–1.34]
Field of view (mm^3^)	117 × 173 × 120	[180–213] × [240–270] × 160	[150–200] × [225–262] × [160–208]
Spatial resolution (mm^3^)	2.00 × 2.00 × 2.00	[1.52–1.88] × [1.67–1.88] × 2.00	[1.53–1.75] × [1.56–1.82] × 2.00
Scan duration (s)	~96	~120	~120

## Data Availability

The computer code and the mechanical phantom data are available upon a reasonable request to the corresponding author.

## Abbreviations

BMI: body mass index
bSSFP: balanced steady-state free precession
CMR: cardiovascular magnetic resonance
DIR: deformable image registration
EDV: end-diastolic volume
EF: ejection fraction
ESV: end-systolic volume
GRE: gradient echo
GT: ground truth
IRB: institutional review board
MRI: magnetic resonance imaging
OPRA: ordered pseudo radial
PVC: premature ventricular contraction
SGD: stochastic gradient descent
SNR: signal-to-noise ratio
SV: stroke volume
SVD: singular value decomposition
TV: total variation
