# Supplementary material for: Continuous Ventricular Volumetric Quantification in Patients with Arrhythmias using Real-Time 3D CMR-MOTUS
Source: ArXiv. 2026 Mar 4:arXiv:2603.04233v1. Preprint. [Version 1] (PMC12976926)
Supplement: Supplement 1 [file NIHPP2603.04233v1-supplement-1.pdf]

Supplementary Information

subject_1.mp4

Supplementary Figure 1: Data in figure is from subject 1. The figure shows a video of the CMR-MOTUS reconstruction, which is a reference image warped with the reconstruction real-time motion fields that are overlaid as green vectors. The segmentation of the left ventricle blood pool is propagated with these motion fields to obtain the real-time ventricular volume .

subject_2.mp4

Supplementary Figure 2: Data in figure is from subject 2. The figure shows a video of the CMR-MOTUS reconstruction, which is a reference image warped with the reconstruction real-time motion fields that are overlaid as green vectors. The segmentation of the left ventricle blood pool is propagated with these motion fields to obtain the real-time ventricular volume .

subject_3.mp4

Supplementary Figure 3: Data in figure is from subject 3. The figure shows a video of the CMR-MOTUS reconstruction, which is a reference image warped with the reconstruction real-time motion fields that are overlaid as green vectors. The segmentation of the left ventricle blood pool is propagated with these motion fields to obtain the real-time ventricular volume.

subject_4.mp4

Supplementary Figure 4: Data in figure is from subject 4. The figure shows a video of the CMR-MOTUS reconstruction, which is a reference image warped with the reconstruction real-time motion fields that are overlaid as green vectors. The segmentation of the left ventricle blood pool is propagated with these motion fields to obtain the real-time ventricular volume.

subject_5.mp4

Supplementary Figure 5: Data in figure is from subject 5 with premature ventricular contractions (PVC). The figure shows a video of the CMR-MOTUS reconstruction, which is a reference image warped with the reconstruction real-time motion fields that are overlaid as green vectors. The segmentation of the left ventricle blood pool is propagated with these motion fields to obtain the real-time ventricular volume .

subject_6.mp4

Supplementary Figure 6: Data in figure is from subject 6 with premature ventricular contractions (PVC). The figure shows a video of the CMR-MOTUS reconstruction, which is a reference image warped with the reconstruction real-time motion fields that are overlaid as green vectors. The segmentation of the left ventricle blood pool is propagated with these motion fields to obtain the real-time ventricular volume.

subject_7.mp4

Supplementary Figure 7: Data in figure is from subject 7 with premature ventricular contractions (PVC). The figure shows a video of the CMR-MOTUS reconstruction, which is a reference image warped with the reconstruction real-time motion fields that are overlaid as green vectors. The segmentation of the left ventricle blood pool is propagated with these motion fields to obtain the real-time ventricular volume.

subject_8.mp4

Supplementary Figure 8: Data in figure is from subject 8 with premature ventricular contractions (PVC). The figure shows a video of the CMR-MOTUS reconstruction, which is a reference image warped with the reconstruction real-time motion fields that are overlaid as green vectors. The segmentation of the left ventricle blood pool is propagated with these motion fields to obtain the real-time ventricular volume.
